# Evaluating the Appropriateness, Consistency, and Readability of ChatGPT in Critical Care Recommendations

**DOI:** 10.1177/08850666241267871

**Published:** 2024-08-08

**Authors:** Kaan Y. Balta, Arshia P. Javidan, Eric Walser, Robert Arntfield, Ross Prager

**Affiliations:** 1 70384 Schulich School of Medicine & Dentistry, Western University, London, Ontario, Canada; 2 Division of Vascular Surgery, Department of Surgery, 7938 University of Toronto, Toronto, Ontario, Canada; 3 Division of Critical Care, London Health Sciences Centre, 6221 Western University, London, Ontario, Canada; 4 Department of Surgery, Trauma Program, London Health Sciences Centre, London, Ontario, Canada

**Keywords:** appropriateness, artificial intelligence, critical care, large language models

## Abstract

**Background:** We assessed 2 versions of the large language model (LLM) ChatGPT—versions 3.5 and 4.0—in generating appropriate, consistent, and readable recommendations on core critical care topics. **Research Question:** How do successive large language models compare in terms of generating appropriate, consistent, and readable recommendations on core critical care topics? **Design and Methods:** A set of 50 LLM-generated responses to clinical questions were evaluated by 2 independent intensivists based on a 5-point Likert scale for appropriateness, consistency, and readability. **Results:** ChatGPT 4.0 showed significantly higher median appropriateness scores compared to ChatGPT 3.5 (4.0 vs 3.0, *P* < .001). However, there was no significant difference in consistency between the 2 versions (40% vs 28%, *P* = 0.291). Readability, assessed by the Flesch-Kincaid Grade Level, was also not significantly different between the 2 models (14.3 vs 14.4, *P* = 0.93). **Interpretation:** Both models produced “hallucinations”—misinformation delivered with high confidence—which highlights the risk of relying on these tools without domain expertise. Despite potential for clinical application, both models lacked consistency producing different results when asked the same question multiple times. The study underscores the need for clinicians to understand the strengths and limitations of LLMs for safe and effective implementation in critical care settings. **Registration:** https://osf.io/8chj7/

## Introduction

Artificial intelligence (AI) has developed rapidly in the past 20 years. Modern machine learning algorithms have been trained on increasingly larger datasets leading to the emergence of sophisticated AI models that recognize and generate human-like text. Particularly, since the public release of OpenAI's large language model (LLM), ChatGPT, in November 2022, there has been a significant adoption and discussion about its use in medicine. ChatGPT has been studied in the context of medical education, passing all 3 United States Medical Licensing Examinations (USMLEs), in academic writing, and in generating and assisting in diagnostic decision making for radiology reports.^1–5^ In the critical care literature evaluating LLMs remains an area of active discussion.^6,7^

A growing area of LLM research is exploring its ability to provide medical recommendations. ChatGPT's responses have been evaluated for the appropriateness of its antimicrobial stewardship, breast cancer prevention and screening recommendations, and cardiovascular disease prevention recommendations.^8–10^ As the LLMs are iterated upon and improved, so do their ability to provide accurate and contextually appropriate, coherent responses. We expect their recommendations to become more appropriate as new training algorithms are developed and larger or more curated datasets are used to train the models.^11,12^

As the use of LLMs by patients and public increases, so too will the need to understand the limitations of these models and the medical recommendations that they provide. Currently, LLMs can produce inaccurate information, however, one notable trait they share is the confidence and commitment to factual inaccuracy.^6^ These “hallucinations” as they are termed, could be dangerous if relied on by patients without medical knowledge, or even by medical professionals without domain knowledge.

The ability of LLMs to provide appropriate, consistent, and readable recommendations in critical care is unclear. The purpose of this study is to assess the ability of an LLM to provide recommendations about core critical care topics; and to compare the relative ability of 2 iterative generations of the LLM. Comparing these generations, we aim to benchmark progress in this field. Additionally, this research will help current clinicians understand limitations of LLMs in their current form, to help avoid inadvertent misinformation.

## Methods

### Ethics Approval

Ethics approval for research not involving human subjects is not required at our institutions.

### Protocol Registration

This protocol was registered on Open Science Framework prior to initiation of data collection: https://osf.io/8chj7/.

### Study Design

We performed a cross-sectional comparative study to evaluate the appropriateness and readability of critical care recommendations generated by LLMs (ChatGPT 3.5 and ChatGPT 4.0, Open AI, https://chat.openai.com/). A predetermined set of clinical questions relating to critical care medicine was developed by 2 intensivists, presented to the AI models, and the resulting recommendations were assessed for appropriateness, consistency, and readability by 2 intensivists independently and in duplicate.

### Question Generation

A set of 50 clinical critical care questions across 5 categories representative of critical care medicine were developed, with questions based on the Textbook of Critical Care, 8th Edition by Jean-Louis Vincent et al. The list of questions is provided in Supplemental Appendix A. The questions do not appear online and were solely synthesized by study authors (EW and RP).

1. *Respiratory*: Airway management, mechanical ventilation, and acute respiratory distress syndrome (ARDS);
2. *Cardiovascular and infectious disease*: Cardiovascular support, hemodynamic monitoring, transfusion, coagulopathy, shock management, sepsis, and infectious disease;
3. *Renal and gastrointestinal*: Acute kidney injury, renal replacement therapy, fluid/electrolyte management, nutritional support, and liver dysfunction;
4. *Central nervous system*: Neurocritical care, management of intracranial hypertension, stroke, analgesia, sedation, and delirium management;
5. *Special considerations*: Ethics, communication, end-of-life care, special populations (eg, obstetrics, pediatrics, and geriatrics), postintensive care syndrome.

### Clinical Appropriateness and Consistency

Each LLM was asked the clinical questions and to provide a recommendation for each question. The questions were asked to ChatGPT between June 10 and 12, 2023 and both models were restricted from accessing internet. The responses were evaluated by 2 critical care clinicians blinded to which model produced them, who scored the recommendations based a 5-point Likert scale for appropriateness developed by Javidan et al for assessing the appropriateness of LLMs.^13^ The 2 scores of the independent reviewers were averaged for each question. The 2 reviewers underwent training on 3 LLM responses not included in the dataset to help standardize scoring.

The responses were scored as the following:

1. *Inappropriate*: The response is grossly factually incorrect, harmful, or overtly contradicts guidelines or reviewer recommendations.
2. *Mostly inappropriate*: The response contains significant inaccuracies, is not comprehensive, lacks critical details, or does not align well with the guidelines or the reviewer's recommendations.
3. *Partially appropriate*: The response provides generally accurate information but could be improved in terms of completeness, depth, or alignment with the guidelines or reviewer's expert recommendations.
4. *Mostly appropriate*: The response is largely accurate, covers most critical information, and aligns with both the guidelines and the reviewer's recommendations, but may have minor omissions or areas that could be further clarified.
5. *Appropriate*: The response is comprehensive, consistent with the guidelines and the reviewer's expert recommendations, and addresses all critical aspects of the clinical question.

### Recommendation Consistency

Consistency is defined as the ability of the LLM to produce similar outputs when asked the same clinical question multiple times. A response was graded as “consistent” if it did not introduce or omit major information that would change the appropriateness of the response. To assess, the same 50 clinical questions were asked 3 times to the AI models in new unique conversations to assess for consistency. The 2 independent reviewers both had to score the 3 responses as consistent for it to be graded as consistent overall.

### Readability Score

We used a calculator to determine the Flesch-Kincaid Grade level as a metric for the readability of the response.^14^ This scale determines the US grade reading level for the text based on length of sentences and number of syllables per word.

### Sample Size Calculation

Based on our hypothesis that ChatGPT4.0 would score a mean Likert score of 4, and ChatGPT3.5 will score a mean Likert score of 3, assuming a population standard deviation of 1.2 Likert point, power of 0.8 and an alpha of 0.05, we required a sample size of 50 prompts for appropriateness.

### Statistical Analysis

All statistical analysis was performed in SPSS (Version 16.0. Chicago, SPSS Inc). All data was stored in Microsoft Excel (Washington, USA). We reported data as mean with standard deviations, medians with interquartile ranges, or count data with percentages as appropriate. Likert scale data were analyzed using the Mann-Whitney U test to account for the ordinal nature of the data, while categorical data were compared using chi-squared tests.

## Results

There was a significantly higher median appropriateness score for ChatGPT 4.0 compared to ChatGPT 3.5. The Mann-Whitney U test indicated a significant difference between the 2 groups, with ChatGPT 4.0 achieving a median score of 4.0 compared to ChatGPT 3.5's median score of 3.0, U = 531.0, *P* < .001. Interrater reliability for appropriateness showed fair agreement (weighted Cohen's kappa of 0.31). There was no significant difference in the consistency of responses provided by ChatGPT 4.0 and ChatGPT 3.5 (20/50 [40%] vs 14/50 [28%], chi-square statistic = 1.114, *P* = 0.291, df = 1). Readability, as measured by the Flesch-Kincaid Grade Level, was not significantly different between the models (ChatGPT 4.0: 14.3, SD 1.5 vs ChatGPT 3.5: 14.4, SD 1.9, *P* = .93), corresponding to university level text reading. Table 1 provides examples of hallucinations, where the LLM produced contextually inaccurate information. Table 2 provides examples of inconsistent lists of recommendations.

**Table 1.** Answers That Include Hallucinations Indicated by ItalicisedText.

Question	Answer
How does an intensivist decide when a patient is ready for extubation?	An intensivist, who is a specialist in critical care medicine, typically decides when a patient is ready for extubation based on several factors, including the patient's clinical status, their level of consciousness, and their ability to breathe on their own. Some common criteria that intensivists use to determine if a patient is ready for extubation include: 1. Stable vital signs 2. Spontaneous breathing 3. Adequate cough reflex 4. Adequate level of consciousness 5. Adequate gas exchange 6. Stable respiratory rate Once the intensivist determines that the patient meets these criteria, they may perform a SBT to assess the patient's ability to breathe on their own. *This involves temporarily removing the patient from the ventilator and providing them with supplemental oxygen, either through a mask or nasal cannula*. The patient's respiratory status is then monitored closely to ensure that they are able to maintain adequate oxygenation and ventilation without assistance. If the patient passes the SBT, the intensivist may proceed with extubation.
What clinical and examination features help determine a patient's “volume status” in the ICU?	Determining a patient's volume status in the ICU involves a comprehensive assessment of various clinical and examination features. Some of the essential features that can help determine a patient's volume status include: 1. Vital signs: The patient's vital signs, including blood pressure, heart rate, respiratory rate, and oxygen saturation, can provide information on the patient's volume status. Low blood pressure, increased heart rate, and reduced oxygen saturation levels may indicate hypovolemia. 2. Fluid balance: A patient's fluid balance is assessed by monitoring their input and output, including urine output, sweat, and stool output, as well as their fluid intake. A negative fluid balance suggests dehydration or hypovolemia, while a positive fluid balance indicates fluid overload. 3. Physical examination: A physical examination may reveal signs of volume overload, such as elevated jugular venous pressure, edema, or ascites, which suggests fluid accumulation. Conversely, physical examination may also reveal signs of hypovolemia, such as dry mucous membranes, decreased skin turgor, and sunken eyes. 4. Laboratory tests: Laboratory tests such as serum sodium, BUN, creatinine, and hematocrit can help determine a patient's volume status. An increased serum sodium level or hematocrit may indicate hypovolemia, while a low BUN level may indicate fluid overload. 5. Echocardiography: Echocardiography can provide valuable information about cardiac function and help determine a patient's volume status. *A low ejection fraction or reduced cardiac output may suggest hypovolemia, while a high ejection fraction or increased cardiac output may indicate fluid overload*. In summary, determining a patient's volume status in the ICU requires a combination of various clinical and examination features. It is crucial to assess the patient's fluid balance, vital signs, physical examination, laboratory tests, and echocardiography to determine their volume status accurately.
What are the first-line, second-line, and third-line therapies for increased ICP?	Increased ICP can be a medical emergency and requires prompt intervention. The treatment of increased ICP typically involves a multimodal approach that includes medical and surgical management. The choice of therapy depends on the underlying cause and severity of the increased ICP. Here are the first-line, second-line, and third-line therapies for increased ICP: First-line therapy: 1. Head of bed elevation: Elevating the head of the bed to 30 degrees can help reduce ICP. 2. Hyperventilation: Hyperventilation is used to reduce arterial carbon dioxide levels and cerebral blood flow, thereby decreasing ICP. 3. Sedation and analgesia: These medications can help reduce cerebral metabolic demand and lower ICP. 4. Mannitol or hypertonic saline: These osmotic agents are used to reduce cerebral edema and lower ICP. Second-line therapy: 1. Barbiturates: Barbiturates are used as an adjunct to other therapies to further decrease cerebral metabolic demand and lower ICP. 2. Decompressive craniectomy: This surgical procedure involves the removal of a portion of the skull to allow for brain swelling and reduce ICP. 3. Hypothermia: Hypothermia is used to reduce cerebral metabolic demand and lower ICP. Third-line therapy: 1. Ventriculostomy: This surgical procedure involves placing a catheter into the brain's ventricles to drain CSF and reduce ICP. 2. *ECMO: ECMO is used to support respiratory and circulatory function in cases of refractory ICP*. 3. *Deep brain stimulation: This treatment involves the implantation of electrodes in specific regions of the brain to modulate neuronal activity and reduce ICP*. It's important to note that the management of increased ICP should be individualized to the patient's specific needs and underlying cause. Therefore, treatment plans may vary among patients.

Abbreviations: BUN, blood urea nitrogen; CSF, cerebrospinal fluid; ECMO, extracorporeal membrane oxygenation; ICP, intracranial pressure; ICU, intensive care unit; SBT, spontaneous breathing trial.

**Table 2.** Consistency When Answering in Triplicate to the Question: What Strategies Exist to Optimize PEEP for Patients with ARDS?

List Item	Answer 1	Answer 2	Answer 3
Individualized approach	✓	✓	✓
Recruitment maneuvers	✓	✓	✓
Titrate PEEP based on oxygenation	✓	✓	✓
Esophageal pressure monitoring	✓	✓	✓
Monitoring ventilatory parameters	✗	✓	✗
Use lung ultrasound	✓	✗	✗
Drive pressure optimization	✗	✓	✓
Assess respiratory system compliance	✗	✓	✗

Abbreviations: ARDS, acute respiratory distress syndrome; PEEP, positive end expiratory pressure.

## Discussion

Medical professionals, patients, and families all have equal access to these public technologies and in our present study we have analyzed their performance without coaching for clinical appropriateness, consistency, and readability. ChatGPT 4.0 produced more appropriate recommendations than ChatGPT 3.5 for critical care scenarios, however, despite the statistically significant improvement between model iterations, Chat GPT 4.0 has yet to meet the complex and nuanced demands of critical care. Furthermore, both models struggle to produce consistent results and produced potentially harmful information. Nonetheless, the iterative improvement between the 2 models helps illustrate the rapid rate of innovation for LLMs and forecasts their potential in a technology driven field like critical care medicine.

Despite iterative improvement in clinical appropriateness of the ChatGPT recommendations, an important pitfall for all LLMs at this stage is the possibility of producing “hallucinations”—confidently conveyed but factually incorrect recommendations.^15^ Hallucinated responses superficially seem accurate given the high degree of conviction with which they are delivered by the LLM, however, the content can be inaccurate, and potentially dangerous. This phenomenon has been well documented in medical and nonmedical applications of LLMs.^16,17^ In our dataset, we identified these hallucinated answers to be primarily driven by inaccurate medical knowledge presented as concrete fact. For example, in Table 1, when asked how an intensivist evaluates a patient's readiness for extubation, a spontaneous breathing trial (SBT) is included among the criteria. The SBT is then defined as removing a patient from the ventilator and providing supplemental oxygen via mask or nasal cannula. If these instructions were to be followed it would be entirely inappropriate. To overcome this limitation, the user must be an expert in the domain of the question, or they can easily be mistaken into falsely accepting the recommendation, given the significant level of conviction with which the information is usually conveyed. This is challenging for clinicians who wish to use LLMs as a medical resource in clinical practice, as people typically search for information in domains that they are not experts in.

In addition to potentially inappropriate recommendations, the consistency across both LLMs was poor. This suggests that if used clinically, LLMs in their current form, may omit potentially important information. For example, when asked what strategies exist to optimize positive end expiratory pressure (PEEP) for patients with ARDS, the LLM accurately recommends esophageal pressure monitoring and titrating PEEP based on oxygenation however only once mentioned titrating based on respiratory system compliance. This implies that even if the results are accurate, they may not be adequately comprehensive with a single prompt.

If used by patients or families, readability is also a challenge for LLMs, with the unprompted responses produced at university reading level that may not be accessible for nonmedical professionals. The difficulty comprehending these responses may further lead to disinformation based on false assumptions. Although LLMs can be specifically asked to produce more readable, accessible, and jargon-free text at a certain reading level (appropriate for patients for example), the accuracy in this content is still unknown.

The improvement in appropriateness between ChatGPT 3.5 and 4.0 already illustrates the strides being made in this field, forecasting a rapidly evolving landscape in the years to come. Yet, in their current forms, they continue to fall short with appropriateness and consistency therefore limiting their clinical use. Nonetheless, they remain available publicly for patients and families to use. Understanding the pitfalls of LLMs in the context of critical care can help intensivists navigate these discussions. There remains room for patients or families to be inadvertently misdirected by this technology by identifying and subsequently advocating for unnecessary testing, invasive procedures, and inappropriate goals of care. In this potential scenario, it may further damage the patient–physician relationship.

In medicine, the opportunities for AIs to improve patient care are innumerable: improving diagnosis from medical imaging, creating patient resources, and reducing human error.^18–20^ Like most technologies, successful implementation depends not only on the technology itself, but an adequate understanding from the clinicians using it. The pandora's box of generative AI has been opened. This public technology may be used by clinicians, colleagues, learners, patients, or families and as the trajectory of improvement continues, the contemporary intensivist must understand and navigate these advancements to leverage their full potential while mitigating risks. This requires continuous education and vigilance to balance innovation with patient safety, data privacy, and ethical considerations, even if these technologies begin reaching clinician level expertise.

## Limitations

There are several notable limitations of this study. Firstly, the questions asked to the LLM did not cover all aspects of critical care, and the limited sample size makes sensitivity analysis of each domain impossible. Additionally, the LLMs were trained on data up until 2021, potentially leading to some recommendations being slightly out of date compared with the most contemporary practice. Finally, the ChatGPT LLMs were chosen given their popularity in recent months, however, there are other commonly used LLMs like Google Bard, scite.ai, and Meta's AI Llama 2—it is unclear how these results generalize to other LLMs.^21–23^

## Conclusion and Next Steps

Generative AI and LLMs have found the spotlight on the center stage of innovation. Additional research is needed to understand how to implement LLMs into clinical practice, and how to ensure safeguards are in place to protect from misinformation.

## Supplemental Material

sj-docx-1-jic-10.1177_08850666241267871 - Supplemental material for Evaluating the Appropriateness, Consistency, and Readability of ChatGPT in Critical Care Recommendations Supplemental material, sj-docx-1-jic-10.1177_08850666241267871 for Evaluating the Appropriateness, Consistency, and Readability of ChatGPT in Critical Care Recommendations by Kaan Y. Balta, Arshia P. Javidan, Eric Walser, Robert Arntfield and Ross Prager in Journal of Intensive Care Medicine
