# Supplementary material for: Evaluating the Appropriateness, Consistency, and Readability of ChatGPT in Critical Care Recommendations
Source: J Intensive Care Med. 2024 Aug 8;40(2):184–90. doi: 10.1177/08850666241267871 (PMC11639400; doi:10.1177/08850666241267871)
Supplement: sj-docx-1-jic-10.1177_08850666241267871 - Supplemental material for Evaluating the Appropriateness, Consistency, and Readability of ChatGPT in Critical Care Recommendations [file sj-docx-1-jic-10.1177_08850666241267871.docx]

**APPENDIX**

**Table A1: All questions asked to large language models organized into 5 categories representative of critical care topics.**

| **Question** | **Prompt** |
| --- | --- |
| **Respiratory: Airway management, mechanical ventilation, and acute respiratory distress syndrome** | |
| 1 | What strategies exist to optimize positive end expiratory pressure (PEEP) for patients with ARDS? |
| 2 | What are evidenced based therapies that reduce mortality in acute respiratory distress syndrome? |
| 3 | For a patient with severe asthma who is intubated for status asthmaticus, what is the optimal approach to ventilating the patient? |
| 4 | How does an intensivist decide when a patient is ready for extubation? |
| 5 | What demographic, clinical, and physiological factors contribute to a potentially difficult airway? |
| 6 | A patient is being mechanically ventilated and suddenly develops increased peak airway pressures: what is the diagnostic approach to this patient and what are 5 potential causes? |
| 7 | What are evidence based indications for non-invasive ventilation (CPAP or BIPAP) for critically ill patients? |
| 8 | What factors go into the decision to perform a tracheostomy on a patient requiring mechanical ventilation? |
| 9 | What is the pathway for liberating a patient’s tracheostomy (decannulation) when it was done for a patient with ARDS who is slow to wean from mechanical ventilation? |
| 10 | For a patient with severe ARDS who is worsening despite maximal support on invasive mechanical ventilation, what rescue options exist and how do clinicians decide which to implement? |
| **Cardiovascular & Infectious Disease: Cardiovascular support, hemodynamic monitoring, transfusion, coagulopathy, shock management, sepsis, and infectious disease.** | |
| 11 | What factors on history, examination, and investigations can help distinguish between distributive and obstructive shock? |
| 12 | What are the indications for arterial line insertion for blood pressure monitoring in the intensive care unit? |
| 13 | What are the potential benefits and harms from the administration of intravenous fluids? |
| 14 | What are appropriate options for vasoactive medications to support blood pressure in the intensive care unit, and what consideration are there for choosing an agent? |
| 15 | What is the optimal ratio of blood products (packed red blood cells, plasma, and platelets) for transfusion during a massive hemorrhage protocol? What other drugs should be given during a massive hemorrhage protocol? |
| 16 | What interventions should be completed within 6 hours of the diagnosis of sepsis? |
| 17 | What factors determine the dosing of antibiotics in sepsis, particularly with respect to the loading dose, and then maintenance doses? |
| 18 | What are the bloodwork abnormalities most commonly associated with disseminated intravascular coagulopathy? |
| 19 | What are important considerations with respect to the differential diagnosis for a patient with “fever in the returning traveller?” |
| 20 | What are the indications and contraindications for extracorporeal membrane oxygenation in severe cardiogenic shock? |
| **Renal & Gastrointestinal:** Acute kidney injury, renal replacement therapy, fluid/electrolyte management, nutritional support, and liver dysfunction | |
| 21 | What features distinguish between pre-renal, renal, and post-renal aetiologies of acute kidney injury in the intensive care unit? |
| 22 | What clinical features can determine whether intermittent hemodialysis compared with continuous renal replacement therapy are the most appropriate form of renal replacement therapy in the intensive care unit? |
| 23 | What patient and clinical factors are important when considering the rate of correction of severe hypernatremia? What is a target sodium correction over 24 hours for a patient with a sodium of 110? |
| 24 | What are the benefits, indications, and contraindications for early enteral nutrition in critically ill patients? |
| 25 | What are first line, second line, and third line therapies for managing acute liver failure? |
| 26 | What is the diagnostic approach, including differential diagnosis, for a patient in the intensive care unit with acute liver failure? |
| 27 | How should the presence and severity of liver cirrhosis impact discussions with family about the prognosis of critical illness? |
| 28 | What clinical and examination features help determine a patient’s ‘volume status’ in the intensive care unit? |
| 29 | What are the diagnostic and therapeutic considerations when managing a patient with severe colitis in the intensive care unit, particularly with respect to surgical intervention? |
| 30 | What are the benefits, risks, and alternatives of total parenteral nutrition for critically ill patients? |
| **CNS:** Neurocritical care, management of intracranial hypertension, stroke, analgesia, sedation, and delirium management | |
| 31 | What are the first line, second line, and third line therapies for increased intracranial pressure? |
| 32 | What are the indications and contraindications for invasive ICP monitoring of critically ill patients? |
| 33 | What is the therapeutic approach to a hyperacute (<4.5 hours from symptom onset) stroke? |
| 34 | What are best practices with respect to analgesia and sedation in the intensive care unit? |
| 35 | What are evidence based strategies for assessing delirium in the intensive care unit? |
| 36 | What is the therapeutic approach to the management of refractory status epilepticus in the intensive care unit? |
| 37 | What is the diagnostic approach to the workup of altered mental status? |
| 38 | What are the diagnostic considerations for a patient with prolonged mechanical ventilation who does not wake when sedation is turned off? |
| 39 | What clinical factors can be used to determine if a patient with neuromuscular weakness (e.g. guillan barre syndrome) requires intubation? |
| 40 | What features suggest a patient as a metabolic encephalopathy compared with a structural cause of decreased level of consciousness in the intensive care unit? |
| **Special Considerations: Ethics, communication, end-of-life care, special populations (e.g., obstetrics, geriatrics), post-intensive care syndrome, rehabilitation** | |
| 41 | What are important factors to consider when disclosing a medical error to the family members of a patient in the intensive care unit? |
| 42 | What ethical considerations exist when a family member who is a substitute decision maker is making decisions based on their own values, and not that of the known values of the patient? |
| 43 | What are the ethical implications of providing futile medical care (medical care for patients that will die in the intensive care unit with or without treatment)? |
| 44 | What are pregnancy-specific considerations when caring for a critically ill patient in their third trimester? |
| 45 | What clinical or demographic factors help prognostic patients with advanced age in the intensive care unit? |
| 46 | What is post-intensive care syndrome, and what strategies can be employed either during or after the intensive care unit admission to reduce its burden and impact on patients and families? |
| 47 | What are important considerations with respect to communication and language when disclosing difficult prognoses to family members in the intensive care unit? |
| 48 | What are potential strategies when dealing with family members of patients in the intensive care unit who disagree with members of the treating team? |
| 49 | What are important diagnostic (including differential diagnoses) and therapeutic considerations for a pregnant patient with seizures? |
| 50 | What factors should be considered prior to transporting a critically ill trauma patient that is mechanically ventilated in a fixed wing air-craft for 3 hours? |
